# Assessing Medical Students’ Preferences for Rural Internships Using a Discrete Choice Experiment: A Case Study of Medical Students in a Public University in the Western Cape

**DOI:** 10.3390/ijerph20206913

**Published:** 2023-10-13

**Authors:** Maria Jose, Amarech Obse, Mark Zuidgeest, Olufunke Alaba

**Affiliations:** 1 School of Public Health, Faculty of Health Sciences, University of Cape Town, Cape Town 7701, South Africa; ag.obse@uct.ac.za (A.O.); olufunke.alaba@uct.ac.za (O.A.); 2 Department of Civil Engineering, Centre for Transport Studies, University of Cape Town, Cape Town 7701, South Africa; mark.zuidgeest@uct.ac.za

**Keywords:** medical students, rural internship, careers, decision-making, health economics, human resources, incentives, labour market, developing countries, occupational health

## Abstract

As new graduates are crucial in providing healthcare services in rural areas, this study aimed to identify and describe the rural facility attributes that attract medical students to apply for rural internships. A literature review and focus groups informed a discrete choice experiment conducted amongst graduating medical students at one public university in South Africa. One main effect using a mixed logit model and another main effect plus interaction model was estimated. Females (130/66.33%) of urban origin (176/89.80%) with undergraduate exposure to rural facilities (110/56.12%) were the majority. The main effects only model showed advanced practical experience, hospital safety, correctly fitting personal protective equipment, and the availability of basic resources were the strongest predictors of rural internship uptake. Respondents were willing to forgo 66% of rural allowance (ZAR 2645.92, 95% CI: 1345.90; 3945.94) for a facility offering advanced practical experience. In contrast, increased rural allowance and housing provision were weak predictors of rural work uptake. Based on the interaction model, females and those not intending to specialise preferred hospital safety compared to advanced practical experience. To improve internship recruitment, rural facility managers should provide staff with supervision, safety, and protection from occupational exposure to contractible illnesses.

## 1. Introduction

The health workforce is a critical building block of a functional health system and a determinant of health service coverage and the attainment of the highest possible standard of health [1,2,3,4]. The lack of adequate skilled personnel in rural areas has been attributed as the top limiting factor to the scale-up of health interventions such as life-saving anti-retroviral treatment and the improvement of maternal and child health outcomes [5].

Rural medical practice is challenging due to social and cultural isolation, lack of infrastructure, transport, electricity, and telecommunications, and restricted access to goods and services [6]. The South African National Department of Health’s (NDOH) strategies for rural doctor recruitment include recruiting rural-origin students to be trained in Cuba on condition of fixed-term mandatory rural service and the provision of on-site housing which is both expensive and time-consuming to maintain [7]. South African medical doctors are trained in undergraduate medical schools (either in South Africa or Cuba), followed by a two-year compulsory internship at an approved government hospital and an additional one-year mandatory community service before they can be certified for independent practice [7]. The training covers a range of clinical disciplines under the supervision of more senior medical personnel. The minimum recommended doctor–population ratio for middle-income countries, such as South Africa (SA), is 18 doctors per 10,000 people [8], but in 2017, the number of medical doctors per 10,000 population in SA was only 9.1 [9]. Only 2.9% of doctors in the SA public sector practice medicine at rural facilities, where an estimated 33% of the population live [10,11]. The doctor shortage is both an absolute as well as a relative issue, as there is an unequal division of doctors along public–private lines, provincial lines, rural–urban lines, poor–wealthy lines, and state-dependent medically insured lines [12]. The overall distribution of public sector post-internship medical posts in South Africa is approximately 75% urban and 25% rural [13]. Despite a desperate need for health workers, in 2003, there was a 31% vacancy rate in the SA public health sector [5]. In 2010, there were 10,860 unfilled public sector medical doctor posts with 46.5% of these in a rural province (Limpopo) compared with 10.2% in an urban province (Gauteng) [14].

Considering the experience of the first five years of practice after graduation as a medical doctor is critical for retention in practicing medicine, there is a need to study the preference of rural internship choices as these can influence further career choices [15]. Studies found that between 6.6 and 45% of newly graduated doctors planned to leave medicine, citing a lack of equipment at facilities and unbearable workload as push factors in the South African public sector [16,17]. During the COVID-19 pandemic, a key concern was protecting health workers from occupational exposure as well as providing supervision and support to prevent burnout. A qualitative cross-sectional study showed that medical interns were motivated to choose an internship based on the proximity of the facility location to family and the fulfilment of their provincial bursary obligations; however, that research was not investigating the preferences for rural facilities specifically [17]. A systematic review found children and/or partners’ integration into rural communities as key to health worker retention [18].

The decision-making context of a final-year medical student starts with communication from the NDOH requiring them to apply online for their internship placement for the following year and listing the available facilities and number of posts at each. After the NDOH receives all applications, posts are allocated; where there is a mismatch of supply and demand (e.g., too many applications for a specific site and too few posts at that site) some applicants will instead be placed on a “2nd round” list. These applicants will be contacted and informed that they did not receive placement at their original choice of internship site and therefore are provided with alternative placement sites which do have vacant posts. Students in this position are forced to choose from amongst these facilities (often outside of popular urban areas) to complete their mandatory internship training.

Although there is literature describing health worker preferences [19,20,21], there is a dearth of knowledge on the internship preferences of South African medical students when faced with choosing between rural placements. In this context, the primary objective of this paper is to describe the relative valuation of rural hospital characteristics among final-year medical students at one public university in the Western Cape Province of South Africa.

## 2. Materials and Methods

### 2.1. Study Design

This study was conducted in one of the public universities in the Western Cape Province of South Africa. There are two universities in the Western Cape that train medical students, one of which was selected to be the setting for this study. The university was chosen as it contributes 50% of the graduates in the Western Cape, and cost prohibited the study from being expanded to more universities. The study population comprised the entirety of final-year medical students at the selected public university who applied for internship placement in 2019 for commencement of work in 2020 (224 students, of which 200 were SA-trained, and 24 were Cuban-trained). Cuban-trained medical students commenced their internship in the latter half of 2019, whereas their SA-trained counterparts commenced their internship in January 2020.

### 2.2. Discrete Choice Experiment

A discrete choice experiment (DCE) is an attributed-based stated-preference method used to elicit preferences for goods or services [22,23]. In a DCE, respondents are presented with a sequence of hypothetical choice questions described by different attributes and levels to select the most preferred alternatives yielding maximum utility [24,25,26].

### 2.3. Attribute Identification

Attributes are the characteristics of the goods/services in the DCE. Attribute levels are the specific values that describe the various features of attributes [26]. Job attributes that are relevant to healthcare workers were identified from the literature and validated by focus group discussions (FGDs) conducted with the study population to identify seven facility attributes (Table 1). As a first step, three FGD sessions were held with a total of 15 medical students representing the gender and provincial distribution of the class. On average, the FGDs took 60 min per session. All FGDs were conducted in English and recorded with respondents’ consent. The FGDs were transcribed verbatim, and thereafter, a thematic analysis was conducted to identify common attributes. Using the FGD guide, students were probed to discuss their views on working in rural areas and what factors can facilitate the take up of an internship job in rural areas.

Students’ sources of information include their experiences at hospital sites, word of mouth from doctors, or social media contact with fellow students, family members, and more senior medical colleagues in their social circles. The student may also visit potential sites, speak to current staff members at a site, or obtain information on the internet about certain internship sites before deciding. This research informs the student’s set of beliefs regarding the job attributes possessed by each internship site (e.g., based on feedback from a fellow student, the supervision at a hospital is reputed to be very good). Students evaluate the list of hospital options available to them to choose from and evaluate them based on personal, facility, and organisational factors. Students rank their preference of internship sites (i.e., 1st choice, 2nd choice, 3rd choice, etc.). Personal considerations that may influence their decision include family commitments, hobbies, and career intentions.

Attributes from the literature that were dismissed by FGD participants were the proximity of a health facility to children’s schools and work opportunities for spouses. Attributes that were identified through the FGDs were personal protective equipment (PPE) in the form of N95 respirator masks to protect against occupational tuberculosis exposure, practical experience, and seniority of the supervisor.

### 2.4. Questionnaire Design

Using the selected attributes and levels, Sawtooth Software (Sawtooth Software Inc., Sequim, WA, USA) was used to generate D-efficient choice sets which consist of 15 hypothetical job postings. The choice scenarios were binary with generically named ‘Rural Hospital A’ and ‘Rural Hospital B’ alternatives. There was no ‘opt-out’ option to reflect the mandatory nature of the internship process for accreditation. The DCE questionnaire was then piloted with 25 final-year medical students from the preceding graduating class. Based on their feedback, the attribute ‘Occupational Hazard’ was specified to include the level ‘incorrectly fitting masks’; these are prone to air leaks which undermine their effectiveness [27]. The attribute ‘Practical Experience’ was reworded to provide clarity and examples for each of its levels. The levels of the attribute ‘Supervision’ are defined based on seniority, with ‘Medical Officer’ the most junior doctor authorised to practise independently, followed by ‘Registrar’, who is a specialist in training, and finally ‘Consultant’, who is an experienced medical specialist. Rural allowance is presented in local currency, the South African Rand (ZAR). The rural allowance base level of ZAR 4000 is based on the South African internship rural allowance of 20% of the monthly base salary of ZAR 20,000 excluding overtime (exchange rate as of 24 August 2020, ZAR 17.02 = USD 1), and the second level was calculated according to historical wage increases as an 8% increase on the base level [28,29]. The third level is a 20% increase on the base level suggested by FGD participants. The attributes ‘Housing’, ‘Basic Resources’, ‘Practical Experience’, and ‘Hospital Safety’ have two levels each, as described in Table 1.

Each hypothetical scenario (choice set) comprised two unlabelled job postings, i.e., Job A and Job B, also known as a preference pair. For the final discrete choice experiment, 15 choice sets were completed by each participant, and the same version of the questionnaire was completed by all participants. An orthogonal design was deemed inappropriate for the required combinations of attributes, levels, and numbers of profiles; therefore, a D-efficient design was used as it aims to minimise the determinant of the covariance matrix with the assumption that the parameters are zero [21]. The design choice sets were unlabelled with choice sets generically named Rural Hospital A and Rural Hospital B, as labels have been shown to distract respondents from job attributes and thus may diminish the reliability of the estimates of attribute preferences, for example, naming a village or district in which the hospital was situated [21].

**Figure 1:**
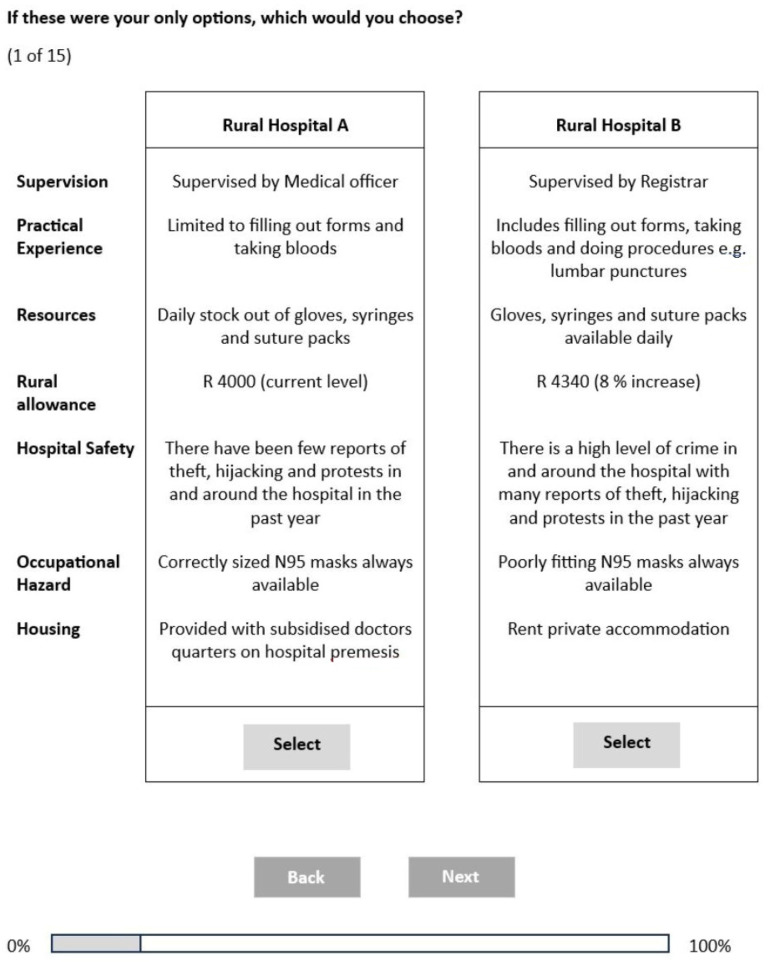
Discrete choice experiment choice set.

The final DCE questionnaire was administered over a one-month period in February 2019. The questionnaire link was sent to the study population via email. It was anonymous and self-administered on devices (laptop/tablet/mobile). It took on average of 20 min to complete (Figure 1). All students have access to computers on campus at computer laboratories, as well as Wi-Fi access. The first author was also in person at class lectures to encourage participation among students and provide refreshments.

### 2.5. Data Analysis

The analysis of the DCE responses followed the random utility theory framework in which individuals are assumed to have an indirect utility for choice alternatives and make choices based on their discrimination capabilities [23]. Given binary choice alternatives of ‘Rural Hospital A’ and ‘Rural Hospital B’ as described by the attributes ($X_{jn}$), students (j) choose the alternative (n) that gives them the highest utility ($U_{jn}$).

(1) $$U_{jn}=V\left(X_{jn},\beta \right)+\varepsilon_{jn}$$

The deterministic part of the utility ($V\left(X_{jn},\beta \right)$), which is observable, is defined as a linear function of the job attribute levels ($X_{jn}$), and the marginal utilities of each attribute level (β) is given by

(2) $$\begin{array}{cc} V\left(X_{jn},\beta \right)=\beta_{0}+ & \beta_{1}\sup\_regist_{jn}+\beta_{2}\sup\_cons_{jn}+\beta_{3}allowance\\ & +\beta_{4}house\_provided_{jn}+\beta_{5}reso\_avai_{jn}+\beta_{6}\exp\_proced_{jn}\\ & +\beta_{7}safety\_good_{jn}+\beta_{8}mask\_poor_{jn}+\beta_{9}mask\_correct_{jn} \end{array}$$

where the variables (X) are defined in Table 1. The attribute ‘Rural Allowance’ is modelled as a continuous variable, while the remaining variables were categorical, and the effects coded. Thus, $\beta_{3}$ indicates a change in utility for a unit change in ‘Rural Allowance’, while the coefficients of the categorical variables capture the effect of the presence of the attribute levels on utility. Two mixed logit (MXL) models, based on 500 Halton draws, were estimated assuming a normal distribution in Stata v14: (i) a main effects only model which is a function of job attributes only, Model 1, and (ii) a main effects plus interaction of attributes with some respondent characteristics (i.e., gender, career aspiration, and prior rural medicine exposure) to explore differences in the valuation of rural internship attributes by sub-population, Model 2.1–2.6. Willingness to pay (WTP) represents the respondent’s preferences for rural health facility attributes in monetary terms. Marginal WTP which indicates how much money a final-year medical student is willing to pay to work at a rural health facility with attribute level (k) in comparison to a facility with the reference attribute level (r) was estimated; this was then expressed in ZAR and as a percentage of the current rural allowance. Given effects coding, for attributes with two levels, marginal WTP was estimated as〖2 x (β〗_k/-β_3), while for attributes with more than two levels, it was calculated as〖β_k-(β〗_r/-β_3), where k ≠ 1 and k ≠ r. The delta method was used to estimate the 95% confidence intervals for the WTP estimates [30].

### 2.6. Ethical Approval

The study was approved by the Ethics Committee and the Student Affairs Departments of the university where the study was undertaken (REF NO 212/2018).

## 3. Results

### 3.1. Demographics

Table 2 presents the respondents’ characteristics. The number of respondents who completed the questionnaire was 193 (86.16%) final-year medical students. The mean age of respondents, 24 years (95% CI 23.65; 23.75), is consistent with an undergraduate, 6-year medical degree. The sample’s female majority, 130 (66.33%), and the distribution of province of origin are reflective of the institute’s admission criteria. The majority of participants came from urban areas, 176 (89.80%), were not married, 183 (93.37%), and did not have child dependents, 193 (98.47%). For respondents who had reported undergraduate exposure to rural medicine, opt-in rural electives, 43 (32.09%), and family medicine rotations, 51 (38.06%), proved most popular. Few respondents were provincial bursary holders, 45 (22.96%), or completed their training in Cuba, 7 (3.57%). One hundred and ninety-two (97.96%) participants intended to complete their internship in SA, with the majority opting to specialise (109/55.61%).

### 3.2. Main Effects Only

Table 3 illustrates the estimation results of the mixed logit model with main effects only (Model 1) and the main effects plus interaction terms for sex (Model 2.1 and 2.2), career intention (Model 2.3 and 2.4), and rural medicine exposure (Model 2.5 and 2.6). All other things constant, a larger mean coefficient translates into a greater relative likelihood of choosing a job alternative with the specific attribute. In the main effects only model, an advanced practical experience (β = 0.919; SE = 0.083) was the most valued attribute, followed by hospital safety (β = 0.770; SE = 0.102), the provision of correctly sized N95 masks (β = 0.718; SE = 0.082), and the availability of basic resources (β = 0.621; SE = 0.072), and these findings were statistically significant. Importantly, the provision of subsidised doctor’s accommodation and rural allowance were among the least valued attributes, though statistically significant. Respondents also preferred job alternatives with consultant supervisors compared to medical officers.

### 3.3. Sub-Group Analysis

The standard deviations of the mean coefficients of attributes in Model 1 are significant at the 1% level, indicating preference heterogeneity among the respondents in relation to these attributes. Therefore, a selection of respondent characteristics (sex, career intention, and rural medicine exposure) was used to further assess heterogeneity in preferences between sub-groups of the respondents using interaction terms (Model 2.1–2.6).

The sub-group analysis by sex (Model 2.1 and 2.2) showed that the top two highly weighted attributes were hospital safety and advanced practical experience for both females and males. However, the third most important attribute for females and males was, respectively, correctly fitting N95 masks and the availability of basic resources. Based on the sub-group analysis by career aspirations (those intending to specialise and those not intending to specialise—Model 2.3 and 2.4), the three most important rural job attributes for those intending to specialise were, respectively, advanced practical experience, hospital safety, and correctly fitting N95 masks. Those not intending to specialise valued hospital safety highest, followed by advanced practical practice and resource availability. The statistically significant findings among respondents without undergraduate rural medicine exposure were that they highly valued the provision of housing and having basic resources available. Among those who had rural medicine exposure, a preference for supervision by consultants and hospital safety were statistically significant.

There was a level of left–right bias present in this sample indicated by a significant Rural Hospital A constant term, 0.375 (*p*-value 0.021). Participant fatigue was ruled to be unlikely by a heteroscedastic conditional logit model which has been used to demonstrate when later choices are not significantly different from earlier choices [31,32].

### 3.4. Willingness to Pay

Respondents’ valuation for their professional development and safety were quantified; they were willing to pay the equivalent of 66.15% in current rural allowance to work in a facility with advanced practical experience compared to a facility which only offered limited practical experience, all other things being equal (Table 4).

## 4. Discussion

This study showed the relative valuation of rural health facility characteristics by medical students in the context where medical students must choose a rural facility among competing rural internship jobs. The medical students from one of the public universities in the Western Cape province of South Africa were chosen as the case study due to the university’s significant contribution to medical graduates annually. The strength of this study is that it contributes to the gap in DCE literature pertaining to medical student’s preferences for internship jobs. These findings can be linked to the wider discourse of aligning human resources for health policy to achieve universal health coverage, which is a key aim of the of the proposed National Health Insurance in South Africa [33].

The most influential attributes to a final-year medical student when considering a rural internship are advanced practical experience, safety, and provisions for protection against occupational hazards. Advanced practical experience is a natural selling point of rural health facilities due to being understaffed and situated far from referral hospitals. Therefore, facility managers of rural facilities should publicise to prospective staff the valuable “hands-on” experience they stand to gain. The finding of advanced practical experience being a highly preferred attribute in the aggregated and sub-group analysis supports the existing literature which found medical students perceived rural internships favourably as an opportunity to have more responsibility and exposure to practical skills [34].

In a sub-group analysis, hospital safety was the most valued attribute of both male and female students. This is a genuine concern in the context of rural facilities, which are often geographically isolated. This finding supports those of Walker and Gilson [35] who documented the experiences of female South African nurses who were victims of crime at their facilities. Encouragingly, the WHO guideline on health workforce recruitment emphasises the safety of healthcare workers in rural and remote facilities as a key recommendation [36].

Personal protective equipment (in this case, N95 masks) is a unique attribute identified in the focus group discussion that has not been studied in other health-worker-recruitment DCE studies. A correctly fitting N95 mask was the third-most important rural internship job attribute in the main model of this study. The importance of personal protective equipment availability among medical students could be interpreted as being due to these students being trained in the Western Cape, which has the highest rates of airborne tuberculosis sampled at public health facilities in South Africa [37]. In a survey among South African medical and physiotherapy students, 49% reported no access to N95 respirators at the health facilities where they were training, which is concerning in the context of HCWs being three times more likely to be at risk of TB disease compared to the general population [37,38]. The ongoing COVID-19 pandemic has highlighted the need for the improved control of airborne pathogens in healthcare settings. Interestingly, a poorly fitting N95 mask was less preferred than having no mask at all by female respondents, but this was marginal (10%) and therefore cannot be generalised for all respondents. At the time of the study, an individual N95 mask cost approximately ZAR 7.76 [39] and are ideally replaced daily, resulting in a monthly cost of approximately ZAR 200 (5% of the rural allowance) at the time of this study’s data collection. However, the increase in demand since the COVID-19 pandemic began has led to rapid price surges with N95 masks trebling in price [40]. Garcia et al. suggest the use of a worker-centred lens for the improved protection of health workers against occupational TB by scale-up screening and raising the awareness of worker’s rights to safe work environments and access to occupational health services [41].

Access to basic resources such as gloves, syringes, and needles was a preference that significantly influenced choices both in the overall and sub-group analyses. In rural facilities that are situated far from medical supply depots, the budgeting and timely procurement of basic resources is vital for the provision of quality healthcare and achieving positive health outcomes [36].

The preference of medical students for consultant supervision places rural facilities at a disadvantage as they are often manned by junior staff. This lack of senior staff may deter graduates intending to specialise from working at rural facilities. Conversely, rural facilities that have consultants should provide them with the responsibility to supervise intern doctors as this is a noted drawcard. This finding supports the existing literature that SA doctors at rural facilities receiving supervision from seniors reported greater levels of job satisfaction and patient care [42]. A study of physicians in Japan highlighted the key role of senior doctors in rural facilities acting as role models to their junior colleagues by demonstrating professionalism and multidisciplinary collaboration, as well as encouraging the practice of reflection to enhance clinical learning [43]. The study by Martin et al. was a cross-sectional questionnaire conducted across ten health professions to explore the experiences of rural student supervisors during the initial phase of the COVID-19 pandemic. They found that not only was the pandemic disruptive to the students’ clinical training, but it also highlighted the need for adequate PPE, as well as effective clinical supervision [44].

The popularity of rural allowance and housing provision as a recruitment strategy is thought to be due to its ability to offset travel expenses, thereby lowering the living expenses associated with living in a rural area [45,46,47,48,49,50]. Although it is found to be statistically significant in the main analysis and within specific sub-groups, neither rural allowance nor housing provision were among the most preferred facility attributes investigated. The main limitation of this study is that it does not compare urban and rural facilities for which the rural allowance was originally proposed as an incentive, and therefore, caution is advised in the interpretation of these findings for broader rural recruitment strategies.

The sub-population analysis highlights heterogeneity in the preference of these job attributes by gender, career aspiration, and rural medicine exposure. Females valued rural allowance and housing provision, which is a finding supported by the literature that found that females were twice more likely to choose a job offer with free housing and were more sensitive to the recruitment effect of rural allowance [48,51]. The career intentions of medical students have been studied in qualitative and quantitative studies [16,52,53] A study of the internship applications trends of Australian medical graduates found that those intending not to specialise preferred rural internship placements compared to those who intended to specialise preferring urban facilities [54]. For the graduate who intends to specialise, rural health facilities can provide the advance practical skills they seek to learn. For those who prefer not to specialise, a rural facility’s safety and resource track record is more influential.

Medical students with rural medicine exposure valued hospital safety highly, reflecting the safety concerns they may have encountered personally or heard about during their time at the rural facility. That medical students without rural medicine exposure preferred being provided with housing contrasts with the existing literature [55]. This could be due to rural-exposed students feeling more confident to organise their own accommodation. Multiple studies, predominantly in Australia, have found that having rural clinical exposure during training was a predictor of future rural clinical practice in the short term [56,57,58].

This study’s findings support the work by Collins and Stevens who advocate that organisations that are recruiting should proactively share their track record of performance on the attributes that potential employees value by making that information readily available in their job postings, social media, and websites [59]. Since students obtain their information about facilities from word-of-mouth referrals, facility managers need to view each current employee as a recruiter and ensure they coherently promote rural practice.

This study uses one graduating class as the sampling frame; although limited to one university, this class is demographically typical to other medical school universities in its age distribution and that females make up the majority of the class [60]. A minority of the sample had either child dependents and/or spouses (1.5% and 6.6%, respectively), which may be why those attributes (schooling for children and work for spouses), which are often cited in the literature, were dismissed during the focus group discussions. Given the stated-preference nature of the experiment and the use of forced-choice scenarios, the overestimation of parameters is a possibility. The small sample size and non-probabilistic sampling strategy, where one public university is selected as a case study, limits the generalisability of results to physicians, although reasonable generalisability can be made for medical students in the province given that the selected university supplies about 50% of medical graduates. Furthermore, this study only assessed preferences between hypothetical competing rural facilities and not preferences of rural facilities in comparison to urban facilities, which is the case. While the sub-group analysis using interaction terms indicated that there is preference heterogeneity across groups, there might also be with-in-group differences which are not presented here. It is argued that these results should be validated by revealed preference data by conducting policy experiments [50]. In reality, minimal information is available about the attributes of a facility, leading jobseekers to base their decisions on rumours of a facility’s reputation; therefore, Robyn et al. encourage greater transparency regarding facility attributes [61].

This study undertaken prior to the COVID-19 pandemic already highlighted the priority that medical students placed on protecting themselves from occupational exposures (in this case, tuberculosis, which is endemic in the region where these students were trained and this study was conducted). The results support the investment case for infection protection and control measures (of which personal protective equipment is but one) as being far less costly and potentially more impactful than further increases in rural allowance or housing provision as recruitment tools.

## 5. Conclusions

Medical students in this study preferred rural internships which would offer a supervised learning environment, safety from physical and occupational hazards, and the provision of basic resources to fulfil their clinical responsibilities. While the study results are not necessarily generalisable to all medical students and/or medical doctors in South Africa, they are consistent with the broad literature on the job preferences of medical doctors. These results can inform policy makers and rural health facility managers in the design of recruitment initiatives that attract underrepresented medical graduates, especially females and those not intending to specialise, through transparent and informative rural facility descriptions. It is hoped that these facility-based incentives would have benefits to both staff and rural health facility users alike.

## Figures and Tables

**Table 1:** Job attributes and levels.

Job Attribute	Level	Variable Name
Supervision	Supervised by Medical Officer [Reference] Supervised by Registrar Supervised by Consultant	-- sup_regist sup_consul
Rural Allowance	ZAR 4000 per month [Reference] ZAR 4340 per month (8% increase) ZAR 4800 per month (20% increase)	allowance
Accommodation	Rent private accommodation [Reference] Provided with subsidised doctors’ quarters on hospital premises	-- house_provided
Resources	Daily stock out of gloves, syringes, and suture packs [Reference] Gloves, syringes, and suture packs are available daily	-- reso_avail
Practical Experience	Limited to filling out forms and taking blood [Reference] Includes filling out forms, taking blood, and performing procedures, e.g., lumbar punctures	-- exp_proced
Hospital Safety	There is a high level of crime in and around the hospital with many reports of theft, hijacking, and protests in the past year [Reference] There have been few reports of theft, hijacking, and protests in and around the hospital in the past year	-- safety_good
Occupational Hazard	No N95 masks available in the hospital [Reference] Poorly fitting N95 masks always available Correctly sized N95 masks always available	-- mask_poor mask_correct

Reference indicates reference level.

**Table 2:** Descriptive statistics of final-year medical students who participated in a discrete choice experiment on rural internship job preferences.

Demographic Characteristics		*n* (%)
Age	Completed years	23.7 (mean)
Gender	Male	63 (31.63)
	Female	130 (66.33)
	Non-conforming	4 (2.04)
Province of origin	Western Cape	72 (36.73)
	Gauteng	47 (23.98)
	North West	3 (1.53)
	Eastern Cape	19 (9.69)
	Kwa-Zulu Natal	38 (19.39)
	Mpumalanga	7 (3.57)
	Limpopo	7 (3.57)
	Northern Cape	3 (1.53)
Area of origin	Rural (village/farm)	14 (7.14)
	Informal settlement (informal structures around town/city)	6 (3.06)
	Urban (formal structure in suburb/township)	176 (89.80)
Marital status	Single	183 (93.37)
	Married	13 (6.63)
Child dependents	Yes	3 (1.53)
	No	193 (98.47)
Undergraduate exposure to rural medicine	Yes	110 (56.12)
	No	86 (43.88)
Rural medicine exposure type	Rural facility placement	8 (5.97)
	An elective at a rural facility	43 (32.09)
	Student society organised rural medicine exposure	32 (23.88)
	Other	51 (38.06)
Provincial bursary holder	Yes	45 (22.96)
	No	151 (77.04)
Cuban-trained student	Yes	7 (3.57)
	No	189 (96.43)
Intention to intern	Yes	192 (97.96)
	No	4 (2.04)
Career intention	General Practice	9 (4.59)
	Specialisation	109 (55.61)
	I don’t know/undecided	70 (35.71)
	Other	4 (2.04)
	Did not intend to complete internship	4 (2.04)

**Table 3:** Mixed logit model results for the discrete choice experiment on rural internship job choices of final-year medical students.

Attribute	Model 1 Mixed Logit Model		Model 2.1 Females	Model 2.2 Males	Model 2.3 Specialise	Model 2.4 Not Specialise	Model 2.5 Undergraduate Rural Medicine Exposure	Model 2.6 No Undergraduate Rural Medicine Exposure
	β (SE)	SD (SE)	β (SE)	β (SE)	β (SE)	β (SE)	β (SE)	β (SE)
Supervision Registrar	0.027 (0.060)	0.385 *** (0.072)	−0.058 (0.085)	0.066 (0.075)	0.001 (0.081)	0.035 (0.127)	0.053 (0.093)	0.111 (0.099)
Supervision Consultant	0.135 * (0.069)	0.323 *** (0.077)	0.137 (0.091)	0.145 * (0.083)	0.254 *** (0.085)	0.128 (0.147)	0.232 *** (0.083)	0.069 (0.124)
								
*(Ref: supervision-medical officer)*								
Rural Allowance	0.001 *** (0.000)	−0.001 *** (0.000)	0.001 *** (0.000)	0.000 (0.000)	0.001 *** (0.000)	0.001 *** (0.000)	0.001 *** (0.000)	0.001 * (0.000)
Housing Provided	0.081 * (0.043)	0.346 *** (0.071)	0.112 * (0.058)	0.029 (0.056)	0.115 * (0.062)	0.119 (0.087)	0.031 (0.058)	0.205 *** (0.067)
*(Ref: private housing)*								
Basic resources available	0.621 *** (0.072)	0.598 *** (0.080)	0.765 *** (0.105)	0.408 *** (0.088)	0.554 *** (0.085)	1.128 *** (0.190)	0.542 *** (0.087)	0.788 *** (0.118)
*(Ref: basic resources not available)*								
Advanced Practical Experience	0.919 *** (0.083)	0.828 *** (0.094)	1.090 *** (0.140)	0.692 *** (0.116)	1.154 *** (0.150)	1.160 *** (0.219)	1.020 *** (0.153)	1.050 *** (0.152)
*(Ref: limited practical experience)*								
Hospital Safe	0.770 *** (0.102)	0.777 *** (0.100)	1.968 *** (0.222)	0.701 *** (0.089)	1.151 *** (0.173)	2.462 *** (0.416)	1.842 *** (0.279)	1.256 *** (0.370)
*(Ref: hospital unsafe)*								
Poorly fitting N95 mask	−0.059 (0.055)	−0.263 *** (0.100)	−0.139 * (0.078)	0.044 (0.070)	−0.022 (0.078)	−0.087 (0.125)	−0.048 (0.075)	−0.069 (0.099)
Correctly fitting N95 mask	0.718 *** (0.082)	−0.456 *** (0.087)	0.832 *** (0.122)	0.294 *** (0.083)	0.802 *** (0.109)	0.875 *** (0.207)	0.703 *** (0.100)	0.858 *** (0.158)
*(Ref: No face mask)*								
No. of Observations	5790		1890	3900	3270	2520	3300	2490
Log Likelihood	−1338.69		−1523.99	−1742.70	−1627.42	−1737.65	−1610.90	−1714.84
Wald chi-squared	200.34		126.12	108.53	97.08	58.60	102.61	102.22
Prob > chi-square	0.0000		0.0000	0.0000	0.0000	0.0000	0.0000	0.0000

***, * significant at 99% and 90%, respectively. Italics indicate a reference level.

**Table 4:** Willingness-to-pay estimates.

Attribute	WTP ZAR Relative to Base (95% CI)	% of Current Rural Allowance
Supervision by registrar	271.45 (−73.79; 616.68)	6.78
Supervision by consultant	427.57 (69.51; 785.63)	10.70
Provision of housing	233.61 (−22.19; 489.41)	5.83
Daily availability of basic resources	1787.13 (915.82; 2658.44)	44.68
Advanced practical experience	2645.92 (1345.90; 3945.94)	66.15
Limited physical threats in and around the facility	2214.92 (1194.74; 3235.11)	55.38
Poorly fitting N95 mask	862.93 (361.32; 1364.54)	21.58
Correctly fitting N95 mask	1980.57 (1074.00; 2887.14)	49.53

## Data Availability

The datasets used during this study are available from the corresponding author on reasonable request.
